# Factors Associated with Smokers Attending More Than One Smoking Cessation Clinic Visit

**DOI:** 10.3390/jcm12237222

**Published:** 2023-11-21

**Authors:** Oh Beom Kwon, Chihoon Jung, Auk Kim, Gihwan Byeon, Seung-Joon Lee, Woo Jin Kim

**Affiliations:** 1 Department of Internal Medicine, Kangwon National University Hospital, Chuncheon 24289, Republic of Korea; obkwon@catholic.ac.kr (O.B.K.); medfman@kangwon.ac.kr (S.-J.L.); 2 Schema Labs Inc. 1, Kangwondaehak-gil, Chuncheon 24341, Republic of Korea; chihoon.jung@cogcom.kr; 3 Department of Computer Science and Engineering, Kangwon National University, Chuncheon 25913, Republic of Korea; kimauk@kangwon.ac.kr; 4 Department of Neuropsychiatry, Kangwon National University Hospital, Chuncheon 24289, Republic of Korea; bjy0421@hanmail.net; 5 Department of Internal Medicine, School of Medicine, Kangwon National University, Chuncheon 25913, Republic of Korea

**Keywords:** smoking cessation, logistic regression, nicotine dependence

## Abstract

Smoking remains a primary cause of cancers, cardiovascular and respiratory diseases and death. Globally, efforts have been made to reduce smoking rates, but the addictive nature of nicotine, a key component of tobacco, makes cessation challenging for smokers. Medical interventions including medical advice and pharmacotherapies are effective methods for smoking cessation. The frequency of medical interventions correlates with success in smoking cessation. This study aims to compare the characteristics of the patients who visited the smoking cessation clinic once with those who visited more than once, in order to identify factors that are associated with repeat clinic visits. A total of 81 patients who have visited the smoking cessation clinic in Kangwon National University Hospital were included. Patients answered the questionnaire at their first visit. If the patient visited only once, the outcome was defined as negative and if the patient visited more than once, the outcome was defined as positive. The proportion of patients who answered “within 5 min” to the Fagerstrom Test for Nicotine Dependence’s (FTND) 1st question and answered “yes” to the FTND’s 6th question was higher in the negative outcome group. In the logistic regression, patients who had withdrawal symptoms previously were associated with positive outcomes (adjusted OR 3.466, 95% CI 1.088–11.034 and *p* value = 0.0354). Withdrawal symptoms during previous attempts were positively related to visiting the clinic more than once.

## 1. Introduction

Smoking has been one of the biggest public health concerns worldwide, contributing to over 8 million deaths annually including 1.3 million non-smokers due to second-hand smoke exposure [1]. In 2020, 22.3% of the world’s population consumed tobacco—36.7% of men and 7.8% of women [2]. In tobacco and tobacco smoke there are group 1 carcinogens including cadmium, phenols and other toxic agents including nicotine, esters, aldehydes, et cetera [3]. In the United States of America (USA), smoking has been linked with most cancers, including those of the lungs, oropharynx, larynx and esophagus, and with non-malignant cardiovascular and respiratory diseases [4]. Beyond this, smoking increases the risk of infection by changing the respiratory tract’s structure and decreasing the immune response [5]. The risk of gastric and duodenal ulcers increases as the packs per year of cigarettes smoked increases [6]. Despite these adverse effects of smoking, it is difficult for smokers to quit smoking due to the fact that nicotine is the primary addictive component of tobacco smoke and causes dependence [7].

The Fagerstrom Test for Nicotine Dependence (FTND) is a widely used questionnaire to evaluate the patient’s intensity of addiction to nicotine [8]. It consists of six items, and among the six items, two items—time to the first cigarette of the day (TTFC) and the number of cigarettes smoked per day (CPD)—are known as the Heaviness of Smoking Index (HSI). TTFC and CPD were important predictors relating to quitting smoking [9]. The Diagnostic and Statistical Manual of Mental Disorders (DSM-5) reported seven common nicotine withdrawal symptoms—irritability, anxiety, difficulty concentrating, increased appetite, restlessness, depressed mood and insomnia [10]. Nicotine dependence, withdrawal symptoms and lack of smoking cessation aids were associated with failure to quit smoking [11]. Yeom H et al. have shown that education level, marital status and the number of counseling sessions were significant factors relating to quitting smoking [12]. Brief advice of up to 5 min from a doctor to encourage smokers to quit smoking is effective and there is correlational evidence that the more intervention time spent with smokers the greater the effect is in terms of quitting smoking [13]. Therefore, in order to increase the probability of smoking cessation, it is important to make smokers visit clinics more frequently. In South Korea, the smoking rate was 31.3% in males and 6.9% in females and the overall smoking rate was 19.3% in 2021, which was ranked fifth among the Organization for Economic Co-operation and Development (OECD) countries [14]. In 2011, the overall smoking rate was 27.1%; the male smoking rate was 47.3% and the female smoking rate was 6.8%. Due to the increase in taxation, cessation programs and smoking-free areas, smoking rates continuously decreased. In 2015, the National Health Insurance Service launched a smoking cessation program in public health centers nationwide. Smokers visit smoking cessation clinics in public health centers six times—the 1st week, 2nd week, 4th week, 6th week and 8th week after the first visit. The 7th and subsequent visits are programmed to prevent a relapse. Smokers pay for the pharmacologic treatment and medical expenses for the 1st and 2nd visits, but if smokers attend from the 3rd session, they get a full refund and receive gifts such as a sphygmomanometer or a blood sugar meter, et cetera. This acts as a strong motivation for smokers to spend more time with health care providers but even with these incentives, the proportion of smokers who visit more than two times is relatively low. Therefore, identifying the factors that prevent smokers from visiting the clinic can aid the smoking cessation program and decrease the overall smoking rate. The general objective of this study was to identify the factors that are associated with more than one smoking cessation clinic visit by smokers. The specific objective of this study was to describe the characteristics of the patients who visited the smoking cessation clinic more than once.

## 2. Materials and Methods

### 2.1. Patients

From 1 January 2021 to 31 May 2023, 84 patients have visited the Kangwon National University Hospital smoking cessation clinic, Chuncheon, Korea. Due to missing data, three patients were excluded. A total of 81 patients were enrolled in this study. Among 81 patients, 70 patients were male and 11 patients were female. The youngest patient was 28 years old and the oldest patient was 87 years old. The number of smoking cessation clinic visits from the patients ranged from 1 visit to 6 visits.

### 2.2. Questionnaire and Variables Definition

Upon their initial visit to the clinic, patients were instructed to complete the Korean version of FTND questionnaire [15], which is shown in Supplementary Material Table S1. Patients were also asked at their first visit to answer Kangwon National University Hospital smoking cessation clinic’s questionnaire regarding the age when they started smoking, packs smoked per year, electronic cigarette (E-cigarettes) usage, alcohol usage, comorbidities, previous smoking cessation attempts, methods they used to quit smoking and the reasons for smoking relapse, the findings from which are presented in Table 1. Answers to the questions were defined as variables. The smoking cessation program used in the study was identical to the program developed by National Health Insurance Service.

### 2.3. Study Design and Statistical Analysis

Outcome was defined by the total number of visits to the Kangwon National University Hospital smoking cessation clinic. If the patients had visited only once, outcome was defined as negative and if the patients had visited more than one time, outcome was defined as positive. Data were expressed as mean ± standard deviation (SD) for continuous variables and number and column percentage for categorical variables. Patients were categorized into two groups by the outcome. Continuous variables were compared using unpaired *t*-test and categorical variables were compared using Chi-squared test (Fisher’s exact test for expected number < 5) between two groups. Logistic regression was performed to analyze variables that were associated with more than one visit and *p* values < 0.05 indicated statistical significance. Statistical analyses were performed using R (version 4.0.2; The R foundation, Vienna, Austria).

## 3. Results

### 3.1. Characteristics of the Patients and Comparison between n = 1 Group and n ≥ 2 Group

The characteristics of the patients are shown in Table 1. We replaced FTND-4, which asks the number of cigarettes smoked per day, with packs smoked per year. The mean of the total number of clinic visits was 1.95 (SD = 1.09). Among 81 patients, 54 patients visited the smoking cessation clinic only once (*n* = 1 group) and 27 patients visited more than twice (*n* ≥ 2 group). The mean of the total number of previous attempts to quit smoking before visiting the smoking cessation clinic was 1.33 (SD = 0.76). The most common reason for failure was stress (54.32%). The second most common reason was lack of self-efficacy (37.03%) and the third reason was withdrawal symptoms (20.99%). The proportion of patients who failed to quit smoking due to withdrawal symptoms was higher in the *n* ≥ 2 group (33.33%) than the *n* = 1 group (14.81%). The FTND score was higher (4.48) in the *n* = 1 group than the *n* ≥ 2 group (3.89) but it was not statistically significant (*p* = 0.1896). As shown in Table 1, FTND-1 and FTND-6 were statistically significant (*p* value < 0.05 and *p* value = 0.003, respectively). In the *n* = 1 group, 50% of the patients smoked within 5 min of waking up and in the *n* ≥ 2 group, 37.04% patients smoked within 5 min of waking up. A total of 50% of the patients in the *n* = 1 group answered that they will smoke even when they are so ill that they have to stay in bed for most of the day, while 14.81% of the patients in the *n* ≥ 2 group answered yes to the same question (FTND-6). A total of 22.22% of the patients in the *n* ≥ 2 group have used smoke cessation acupuncture to quit smoking previously and 1.85% in the *n* = 1 group have used acupuncture previously.

### 3.2. Factors Associated with Number of Visits to the Clinic

The results of the univariable and multivariable logistic regressions are shown in Table 2. In the univariable logistic regression analysis, withdrawal symptoms (unadjusted odds ratio (OR) 2.875, 95% confidence interval (CI): 0.960–8.613 and *p* value = 0.059) and packs smoked per year (unadjusted OR 0.296, 95% CI: 0.082–1.065 and *p* value = 0.0624) had *p* values < 0.2. Since FTND-1 and FTND-2 were significantly different between the groups, the FTND score and the variables with *p* value < 0.2 were entered in the multivariable logistic regression. In the multivariable logistic regression, only the withdrawal symptom remained statistically significant. (adjusted OR 3.466, 95% CI 1.088–11.034 and *p* value = 0.0354).

## 4. Discussion

Among 81 patients who visited the Kangwon National University Hospital’s smoking cessation clinic, 54 patients (66.67%) have visited only once and 27 patients (33.33%) have visited more than one time. A total of 17 patients visited two times, 23 patients visited three times and 4 patients visited more than three times. The smoking cessation program accounts for approximately 41% of the 6-month success rate for quitting smoking [16]. Although the program was proved to be effective for quitting smoking, it is difficult to increase a patient’s compliance, as shown in our study. The majority of the patients visited only once. This was consistent with the previous study which showed that more than half of patients have dropped out the smoking cessation clinic program after one or two visits [17]. The mean of attempts to quit smoking before visiting the clinic was 1.33 times (SD 0.76). The mean of the number of methods to quit smoking was 2.33 (SD 1.36). The majority of the patients (61.73%) tried to quit smoking with self-efficacy without the help of healthcare providers. A total of 33.33% of the patients used pharmacotherapy and 8.64% of the patients used acupuncture and herbal cigarettes.

Answers to FTND-1 and FTND-6, along with the level of usage of herbal cigarettes or acupuncture to quit smoking before visiting the clinic, were significantly different between the *n* = 1 group and the *n* ≥ 2 group. FTND-1 asks how soon the patient wants to smoke after waking up and is a component of HSI, which is an important risk factor for failure to quit smoking. The proportion of the patients who answered “yes” to the question about whether they would smoke even if they were sick and had to stay in bed for the whole day was significantly higher in the *n* = 1 group (50.00%) than the *n* ≥ 2 group (14.81%). More incentives or more interventions should be offered to the patients who replied “within 5 min” and “yes” to these two questions in order to increase the compliance with the smoke cessation program and ultimately lead to success in quitting smoking. The numbers of patients who used herbal cigarettes or acupuncture as a method for quitting smoking before visiting the smoking cessation clinic was significantly higher in the *n* ≥ 2 group (22.22%) than in the *n* = 1 group (1.85%). This might be due to the fact that patients who use these relatively unusual methods to quit smoking have more willingness to quit smoking. However, previous studies showed lack of evidence for herbal cigarettes and acupuncture [18,19]. A total of 20.99% of the patients replied that they failed to quit smoking due to withdrawal symptoms and the proportion was higher in the *n* ≥ 2 group (33.33%) than the *n* = 1 group (14.81%) but it was not statistically significant (*p* = 0.053). This might be due to the relatively small number of patients enrolled in our study. Since HSI is an important predictor for quitting smoking, packs smoked per year was considered as a variable for logistic regression analysis. Since smokers have an average of six to seven attempts to quit smoking before they ultimately succeed in quitting smoking [16], we added the number of previous attempts as a variable. The more attempts they made may represent the willingness of a patient and influence the compliance of a patient.

Withdrawal symptoms at previous attempts, packs smoked per year, number of previous attempts and FTND score were selected as variables for the logistic regression, as shown in Table 2. Patients who had withdrawal symptoms during previous attempts had an adjusted OR of 3.466 greater than the patients who did not have withdrawal symptoms. As shown in Table 1, the majority of the patients did not undergo interventions such as pharmacotherapy and medical counseling at previous attempts. Most of the previous attempts relied on self-efficacy. Patients who had withdrawal symptoms during previous attempts might feel that self-efficacy is not enough to quit smoking and medical intervention is needed to quit smoking. As a result, patients with withdrawal symptoms visited the clinic more than the patients who did not have withdrawal symptoms. However, a previous study has shown that smokers who had withdrawal symptoms when they tried to quit smoking before were less likely to success with smoking cessation [20]. Therefore, clinicians should try to increase the compliance of patients who did not have withdrawal symptoms at their previous attempt and should try to focus on managing withdrawal symptoms for patients who had withdrawal symptoms previously.

More intervention time spent with smokers is associated with smoking cessation [13]. Our study has investigated the factors that are related to the number of visits to the smoking cessation clinic. The more the patient visits the clinic, the higher the probability is that they will quit smoking. Patients who answered “within 5 min” to FTND-1, “yes” to FTND-6 and who did not have withdrawal symptoms during previous attempts should undergo more intensive interventions. This study has two advantages. First, there were previous studies to identify the factors that were associated with smoking cessation but, to the best of our knowledge, there was no study that identified the factors that were associated with the number of smoking cessation clinic visits. Since the number of smoking cessation clinic visits is associated with successful smoking cessation [21], it is crucial for smokers to visit the clinic more often. Our study identified the factors that are associated with the number of visits so that clinicians can treat smokers who are unlikely to visit the clinic more intensively. Second, we have shown that the majority of smokers tried to quit smoking without the help of medical interventions. The key components of successful cessation are combinations of medical counseling and pharmacotherapy [22]. Our study suggests the need for campaigns for smokers that emphasize the importance of medical interventions to help stop smoking. However, our study has several limitations. First, the number of patients was relatively small. Second, the variables that were relied on were the patient’s answers to the questionnaires so there might be some bias. Third, patients might not be able to visit the clinic due to other reasons such as moving to other regions. Fourth, the study was based on cross-sectional data so it was difficult to derive causal relationships. Further longitudinal studies with larger number of patients are needed.

## 5. Conclusions

The proportion of patients who smoked within 5 min of waking up and who are willing to smoke even if they are so ill that they have to stay in bed for the whole day was higher for the group of patients who visited the smoking cessation clinic only once than for the group of patients who visited more than once. In the logistic regression, withdrawal symptoms during previous attempts were positively related to visiting the clinic more than once. (Adjusted OR 3.46 and *p* value = 0.0354.)

## Figures and Tables

**Table 1:** Characteristics of the patients and comparison between *n* = 1 group and *n* ≥ 2 group.

	Total N = 81	*n* = 1 Group N = 54	*n* ≥ 2 Group N = 27	*p*-Value
BMI	24.67 ± 3.84	24.26 ± 3.86	25.50 ± 3.72	0.167
Age	57.94 ± 14.45	58.19 ± 14.62	57.44 ± 14.36	0.829
Male	70	44	26	
Age at first smoking	20.78 ± 5.37	20.19 ± 5.07	21.96 ± 5.83	0.184
Pack years	30.61 ± 24.60	33.47 ± 21.96	24.90 ± 28.79	0.181
E-cigarettes	21 (25.93)	15 (27.78)	6 (22.22)	0.591
Alcohol usage	48 (59.26)	30 (55.56)	18 (66.67)	0.337
Drinking number	1.74 ± 2.18	1.65 ± 2.29	1.93 ± 1.96	0.572
Depression	2 (2.47)	1 (1.85)	1 (3.70)	1.000
Hypertension	30 (37.03)	19 (35.19)	11 (40.75)	0.626
Diabetes	19 (23.46)	13 (24.07)	6 (22.22)	0.853
Dyslipidemia	20 (24.69)	14 (25.93)	6 (22.22)	0.716
Previous attempt	21 (29.93)	14 (25.93)	7(25.93)	1.000
Number of previous attempts	1.33 ± 0.76	1.28 ± 0.74	1.44 ± 0.80	0.369
FTND score	4.28 ± 1.99	4.48 ± 2.06	3.89 ± 1.80	0.190
Total number of visits	1.95 ± 1.09	1.30 ± 0.50	3.26 ± 0.71	
Failure reasons				
Lack of self-efficacy	30 (37.03)	20 (37.04)	10 (37.03)	1.000
Withdrawal symptoms	17 (20.99)	8 (14.81)	9 (33.33)	0.053
Stress	44 (54.32)	29 (53.70)	15 (55.56)	0.875
Friends/peer pressure	8 (9.88)	5 (9.26)	3 (11.11)	1.000
Weight gain	4 (4.94)	2 (3.70)	2 (7.41)	0.596
FTND-1				*p* < 0.001
Within 5 min	37 (45.68)	27 (50.00)	10 (37.04)	
5–30 min	25 (30.86)	13 (24.07)	12 (44.44)	
31–60 min	7 (8.64)	4 (7.41)	3 (11.11)	
Over 6 min	12 (14.81)	10 (18.52)	2 (7.41)	
FTND-2 Number of patients who answered YES	21 (25.93)	15 (27.78)	6 (22.22)	0.590
FTND-3 Number of patients who answered The first of the morning	29 (35.80)	18 (33.33)	11 (40.74)	0.510
FTND-5 Number of patients who answered YES	21 (25.93)	12 (22.22)	9 (33.33)	0.282
FTND-6 Number of patients who answered YES	31 (38.27)	27 (50.00)	4 (14.81)	0.003
Method to quit smoking	2.33 ± 1.36	2.29 ± 1.40	2.37 ± 1.31	0.861
Self-efficacy	50 (61.73)	33 (61.11)	17 (62.96)	0.872
Pharamacotherapy	27 (33.33)	17 (31.48)	10 (37.04)	0.617
Telephone counseling	1 (1.23)	1 (1.85)	0	1.000
Herbal cigarettes/Acupuncture	7 (8.64)	1 (1.85)	6 (22.22)	0.0049

BMI, body mass index; E-cigarettes, electronic cigarettes FTND and Fagerstrom Test for Nicotine Dependence.

**Table 2:** Univariable and multivariable logistic regressions for smoking cessation clinic visits occurring more than two times.

	Univariable Logistic Regression			Multivariable Logistic Regression		
	OR	95% CI	*p* Value	OR	95% CI	*p* Value
Withdrawal symptoms	2.875	(0.960, 8.613)	0.059	3.466	(1.088, 11.034)	0.0354
Pack years	0.296	(0.082, 1.065)	0.0624	0.985	(−0.04, 1.010)	0.241
Number of previous attempts	1.334	(0.727, 2.447)	0.352			
FTND score	0.856	(0.087, 0.672)	0.208	0.903	(0.677, 1.205)	0.490

OR, odds ratio; CI, confidence interval and FTND, Fagerstrom Test for Nicotine Dependence.

## Data Availability

The data presented in this study are available on request from the corresponding author.

## Supplementary Materials

The following supporting information can be downloaded at: https://www.mdpi.com/article/10.3390/jcm12237222/s1, Table S1: Fagerstrom Test for Nicotine Dependence (FTND) Questionanaire.
