# Supplementary material for: Factors Associated with Smokers Attending More Than One Smoking Cessation Clinic Visit
Source: J Clin Med. 2023 Nov 21;12(23):7222. doi: 10.3390/jcm12237222 (PMC10706923; doi:10.3390/jcm12237222)
Supplement: Supplementary file 1 [file jcm-12-07222-s001.zip › jcm-2699499-supplementary.pdf]

Table S1. Fagerstrom Test for Nicotine Dependence (FTND) Questionanaire

No.	Question	Answer
1	How soon after waking up do you smoke your first cigarette?	Within 5 minutes
		Within 6 - 30 minutes
		Within 31 - 60 minutes
		After 60 minutes
2	Do you find it difficult to refrain from smoking in places where it is forbidden?	No
		Yes
3	Which cigarette would you hate most to give up?	The first one in the morning
		All others
4	How many cigarettes per day do you smoke?	10 or less
		11 - 20
		21 - 30
		31 or more
5	Do you smoke more frequently during the first hours after waking than during the rest of the day?	No
		Yes
6	Do you smoke if you are so ill that you are in bed most of the day?	No
		Yes
